# Transmucosal Buprenorphine in the Treatment of Dyspnea: Case Series and Review of the Literature

**DOI:** 10.1089/pmr.2020.0091

**Published:** 2021-01-12

**Authors:** François Blumenfeld-Kouchner, Lisa Bullis, Kathy Koch

**Affiliations:** ^1^ Aurora Medical Group Palliative Care, Milwaukee, Wisconsin, USA.; ^2^ Department of Medicine, School of Medicine and Public Health, University of Wisconsin, Madison, Wisconsin, USA.; ^3^ Advocate Aurora Library, Advocate Aurora Health, Grafton, Wisconsin, USA.

**Keywords:** breathlessness, buprenorphine, case series, dyspnea, opioid analgesics

## Abstract

Full μ-opioid agonists are commonly employed in advanced disease to relieve dyspnea of various etiologies. Although there are ongoing debates and studies regarding the relative efficacy of different agents, a factor limiting more general use is a concern about side effects, in particular respiratory depression. Buprenorphine is a partial μ receptor agonist and a κ-opioid receptor antagonist, with a better safety profile than full μ receptor agonists. We conducted a literature search, which did not reveal any studies looking at the use of buprenorphine to treat dyspnea. We also report a case series of three patients with dyspnea of various etiologies treated with buprenorphine, with an apparent excellent response of dyspnea to treatment, without any significant side effects. Given those results, as well as the potential impact of κ-opioid receptor antagonism on dyspnea-associated anxiety, we conclude that there is a need for controlled studies of buprenorphine against full μ-opioid agonists for the symptomatic treatment of dyspnea in palliative care.

## Introduction

Opioids are commonly used in the palliative treatment of dyspnea of multiple etiologies. Buprenorphine is a μ-opioid receptor (MOR) partial agonist, and a κ-receptor antagonist. It is considered safer than full MOR agonists in terms of respiratory depression risk.^1,2^ Evidence suggests that a limiting factor to the prescription of opioids for dyspnea is a concern about safety and side effects.^3,4^

In addition, although expert opinion considers low-dose full MORs helpful in the treatment of refractory dyspnea, their benefit has been difficult to demonstrate in randomized controlled settings.^5,6^ One reason for this may be that assessment of dyspnea through questionnaires may be underestimating the impact of treatment as patients may increase their activity level to reach their previous breathlessness severity level if the treatment is helpful; it has been suggested that more objective measures, such as use of accelerometers to measure changes in activity, may be better suited to assess treatment effects.^7,8^ Nevertheless, patients seen in palliative care settings often suffer from comorbid pain, also raising concerns about need for higher opioid doses (and hence side effects) than might suffice to treat isolated dyspnea.

Buprenorphine thus represents an intriguing option for the treatment of dyspnea, both for its different actions on MORs compared with traditional opioids and for its intrinsic safety including at higher doses, which could help overcome barriers in prescription, particularly from nonpalliative specialists.

We sought to determine whether buprenorphine had been examined as a treatment for dyspnea. In the absence of previously reported evidence, we submit a case series of patients with dyspnea treated using transmucosal buprenorphine preparations in the setting of advanced chronic obstructive pulmonary disease (COPD) and cancer. Transmucosal formulations of buprenorphine exist, among others, as dissolving films (buccal or sublingual) of either plain buprenorphine at low doses (micrograms) intended for chronic pain management, or as a combination of buprenorphine and naloxone (the latter is intended as a deterrent for misuse through injection and remains inactive when the film is taken through the transmucosal route) intended for treatment of opioid dependence, with buprenorphine typically in milligrams range. Dosing for chronic pain in opioid-naive patients is 75 mcg once daily to every 12 hours, titrated up to 150 mcg every 12 hours. By contrast, dosing of buprenorphine–naloxone for opioid use disorder (OUD) typically starts at 2 mg buprenorphine, with usual range up to 24 mg daily in divided doses.^9^

## Review of the Literature

We conducted a literature search in October 2020 in MEDLINE, PubMed, Cochrane Library, CINAHL, Google Scholar, ClinicalTrials.gov, and EU Clinical Trials Register for English language articles using a keyword search strategy of (dyspnea OR dyspnoea OR breathlessness OR shortness of breath) AND buprenorphine. Reference lists in relevant articles were also reviewed.

No study on the use of buprenorphine for the treatment of dyspnea was found. Seven articles evaluating treatment of pain with buprenorphine mentioned dyspnea. Two publications originating from one randomized, unblinded, and nonplacebo controlled study reported a lack of impact of transdermal buprenorphine on dyspnea as measured by quality-of-life questionnaires.^10,11^ Of note, patients with “symptoms of respiratory insufficiency” were excluded and none of the tested opioids had any effect on dyspnea, which was attributed to low severity of dyspnea in study patients.

Five studies reported dyspnea as an adverse effect of buccal and transdermal buprenorphine used for treatment of chronic noncancer-related pain: two randomized trials reported dyspnea in 1 out of 254 and 0.5% of patients in the buprenorphine group, respectively.^12,13^ One randomized trial reported dyspnea and chest pain in 1 of 36 patients treated with buprenorphine.^14^ The two remaining studies were incorporated in a retrospective analysis concluding that the incidence of dyspnea did not differ significantly between placebo and buprenorphine groups.^15^

## Case Series

We present a series of three patients seen in our palliative care clinics for whom transmucosal (both buccal and sublingual) buprenorphine was prescribed for dyspnea and other symptoms.

All patients provided written informed consent for inclusion in this report. None of the patients experienced adverse effects attributable to buprenorphine, although they all required laxative therapy to prevent constipation.

### Case 1: A 63-year-old woman with emphysema and pulmonary hypertension

This patient was diagnosed with COPD by pulmonary function testing (PFT) in 2017, and was at the time of referral classified as Global Initiative for Chronic Obstructive Lung Disease stage 3D. Moderate pulmonary hypertension was diagnosed by transthoracic echocardiography. She also suffered from chronic pain resulting from a motor vehicle incident in 2014. After her second hospitalization for a COPD exacerbation within a year, she was found to have comorbid severe OUD. She endorsed relief of her dyspnea with heroin use. Given two strong indications for prescription of buprenorphine (chronic pain and OUD), she consented to treatment with sublingual buprenorphine–naloxone and sought to discover whether buprenorphine also improved her breathlessness.

She was eventually titrated to a stable split dose of 8 mg buprenorphine three times daily, and has remained abstinent of other opioids.

After one week of treatment, the patient's family noted that she no longer needed to stop to catch her breath when walking from her bed to the bathroom. In addition, buprenorphine helped with chronic pain that also impacted her activity level, and she was eventually able to enroll in pulmonary rehabilitation.

This patient's experience with buprenorphine and incidental improvement in dyspnea led us to consider use in the following cases.

### Case 2: A 67-year-old woman with asthma and metastatic colorectal cancer

This patient's childhood asthma was well controlled for decades on chronic prednisone, theophylline, and montelukast. PFT in 2017 revealed a moderate obstructive defect. After her cancer diagnosis in early 2020, which included hepatic metastases, her dyspnea on exertion increased dramatically. With no apparent cardiac cause, and a computed tomography angiogram of her chest revealing no embolism and only millimetric peripheral lung nodules, a definitive etiology of her worsening dyspnea could not be ascertained. She developed anemia, but her symptoms did not appear to fluctuate with changes in hemoglobin level.

Owing to prior experiences of oversedation with full MOR agonists, a trial of transmucosal buprenorphine was agreed upon. Improvement of exertional dyspnea was noted within 12 hours of the first dose of 4 mg (sublingual buprenorphine–naloxone formulation), which however caused nausea (no sedation). The dose was then decreased to 1 mg buprenorphine once daily in the morning. Continued effect on dyspnea on exertion was noted, but lasting only until the later afternoon. Buprenorphine was thus increased to 1 mg twice daily just under two weeks after treatment had started.

She measured her functional improvement by noting an increased step count from 2000 steps a day before treatment initiation (limited by dyspnea) to 6500 steps a day within two weeks of treatment initiation. No modifications to her asthma treatments were made during this period.

### Case 3: A 56-year-old man with a neuroendocrine tumor metastatic to thoracic lymph nodes and lung parenchyma

This patient was diagnosed with cancer of the appendix causing intestinal obstruction in mid-2019. He later developed dyspnea on exertion with cough and was found to have thoracic metastases; there was no clinical or imaging evidence of prior or underlying pulmonary or cardiac disease. The patient also suffered from chemotherapy-induced neuropathic pain for which he would occasionally use oxycodone. It was verified that oxycodone administration before activity improved his exertional dyspnea.

Concerns about experienced sedation with oxycodone and importance of maximizing activity level led to an interest in a trial of buprenorphine, given our prior experience in the other cases. Buccal buprenorphine film was initiated at 75 mcg once daily. Within the first two days of treatment, the patient reported improved dyspnea and an increase in his activity tolerance, from completing all his house chores but lacking outside activity to resuming yard work and completing a 1-mile walk. No untoward side effects were noted.

## Conclusion

In this case series, we describe three patients with exertional dyspnea of multifactorial etiology, known to be opioid responsive, who reported improvement in their symptoms without adverse events after initiation of transmucosal buprenorphine. Given these findings, the significant differences in pharmacology between buprenorphine and full MOR agonists (including antagonism at κ-opioid receptors, which may have a role in the modulation of anxiety^16^) and buprenorphine's favorable safety profile, well-designed controlled studies addressing buprenorphine's effect on dyspnea are warranted.

## Abbreviations Used

COPD: chronic obstructive pulmonary disease
MOR: μ-opioid receptor
OUD: opioid use disorder
PFT: pulmonary function testing

## References

[1] Walsh SL, Preston KL, Stitzer ML, et al.: Clinical pharmacology of buprenorphine: Ceiling effects at high doses. Clin Pharmacol Ther 1994;55:569–580 8181201 10.1038/clpt.1994.71

[2] Dahan A, Yassen A, Romberg R, et al.: Buprenorphine induces ceiling in respiratory depression but not in analgesia. Brit J Anaesth 2006;96:627–632 16547090 10.1093/bja/ael051

[3] Hadjiphilippou S, Odogwu S-E, Dand P: Doctors' attitudes towards prescribing opioids for refractory dyspnoea: A single-centred study. BMJ Support Palliat Care 2014;4:190–192 10.1136/bmjspcare-2013-000565 24644207

[4] Pattinson KTS, Rowland MJ, Nickol AH, Quinlan J.: Adverse respiratory effects of opioids for chronic breathlessness: Learning lessons from chronic pain. Eur Respir J 2018;51:1702531 29496786 10.1183/13993003.02531-2017

[5] Verberkt CA, van den Beuken-van Everdingen MHJ, Schols JMGA, et al.: Effect of sustained-release morphine for refractory breathlessness in chronic obstructive pulmonary disease on health status. JAMA Intern Med 2020;180:1306–1314 32804188 10.1001/jamainternmed.2020.3134 PMC7432282

[6] Currow D, Louw S, McCloud P, et al.: Regular, sustained-release morphine for chronic breathlessness: A multicentre, double-blind, randomised, placebo-controlled trial. Thorax 2020;75:50 31558624 10.1136/thoraxjnl-2019-213681

[7] Currow D, Watts GJ, Johnson M, et al.: A pragmatic, phase III, multisite, double-blind, placebo-controlled, parallel-arm, dose increment randomised trial of regular, low-dose extended-release morphine for chronic breathlessness: Breathlessness, exertion and morphine sulfate (BEAMS) study protocol. BMJ Open 2017;7:e018100 10.1136/bmjopen-2017-018100 PMC5726102 28716797

[8] Bade BC, Brooks MC, Nietert SB, et al.: Assessing the correlation between physical activity and quality of life in advanced lung cancer. Integr Cancer Ther 2016;17:73–79 28024420 10.1177/1534735416684016 PMC5647199

[9] Leppert W, Nosek K.: Comparison of the quality of life of cancer patients with pain treated with oral controlled-release morphine and oxycodone and transdermal buprenorphine and fentanyl. Curr Pharm Design 2019;25:3216–3224 10.2174/1381612825666190717091230 31333114

[10] Buprenorphine [version 385.0] and Buprenorphine-naloxone [version 322.0] in UpToDate, Inc. (Lexi-Drugs). UpToDate, Inc. http://www.uptodate.com/contents/buprenorphine-drug-information and https://www.uptodate.com/contents/buprenorphine-and-naloxone-drug-information (Last accessed 1012, 2020)

[11] Nosek K, Leppert W, Nosek H, et al.: A comparison of oral controlled-release morphine and oxycodone with transdermal formulations of buprenorphine and fentanyl in the treatment of severe pain in cancer patients. Drug Des Dev Ther 2017;11:2409–2419 10.2147/DDDT.S141007 PMC5574595 28860712

[12] Gimbel J, Spierings ELH, Katz N, et al.: Efficacy and tolerability of buccal buprenorphine in opioid-experienced patients with moderate to severe chronic low back pain: Results of a phase 3, enriched enrollment, randomized withdrawal study. Pain 2016;157:2517–2526 27434505 10.1097/j.pain.0000000000000670 PMC5065057

[13] Rauck RL, Potts J, Xiang Q, et al. Efficacy and tolerability of buccal buprenorphine in opioid-naive patients with moderate to severe chronic low back pain. Postgrad Med 2015;128:1–11 26634956 10.1080/00325481.2016.1128307

[14] Webster L, Gruener D, Kirby T, et al.: Evaluation of the tolerability of switching patients on chronic full μ-opioid agonist therapy to buccal buprenorphine. Pain Med 2016;17:889–907 10.1093/pm/pnv110 PMC4984426 26917621

[15] Pergolizzi JV, Raffa RB, Marcum Z, et al.: Safety of buprenorphine transdermal system in the management of pain in older adults. Postgrad Med 2016;129:92–101 27929709 10.1080/00325481.2017.1270699

[16] Peters MF, Zacco A, Gordon J, et al.: Identification of short-acting κ-opioid receptor antagonists with anxiolytic-like activity. Eur J Pharmacol 2011;661:27–34 21539838 10.1016/j.ejphar.2011.04.017

